# The Study of Clustering Effects of Behavior Risk Factors in Patients with Metabolic Syndrome in Southern China: A Cross-Sectional Study

**DOI:** 10.1155/2020/6478393

**Published:** 2020-07-01

**Authors:** Huiwu Han, Fan Zheng, Weiwei Dai, Hua Peng, Shi Zhou, Huixia Tian

**Affiliations:** ^1^Xiangya School of Nursing, Central South University, Changsha, China; ^2^Xiangya Hospital at Central South University, Changsha, China; ^3^Health Management Department, Xiangya Hospital at Central South University, Xiangya Road 87#, Changsha, Hunan, China

## Abstract

**Objectives:**

Metabolic syndrome (MetS) is now becoming a serious public health threat. Some behaviors risk factors were considered to be associated with MetS and interacted to adversely affect MetS. However, the clustering effects of behavior risk factors of MetS among Chinese population remain unclear. The aim of this study is to observe the behavior risk factors and their clustering effects of MetS in China.

**Methods:**

A cross-sectional study design was used. Subjects were recruited in the departments of Cardiology Clinic, Endocrine Clinic, and Health Management from March to December 2019. A demographic sheet was designed to collect the demographic and clinical characteristics of the subjects. International Physical Activity Questionnaire-Short was applied to evaluate the level of PA in this study. Other behavior risk factors were observed by the questionnaire. The stepwise logistic regression analysis was performed to identify the determinants of MetS. The multiple logistic regression analysis was used to analyze the clustering effects of behavior risk factors in MetS.

**Results:**

There are a total of 532 eligible subjects (56.6% females; mean age was 48.4 ± 15.3 years), and approximately 33.3% were diagnosed as MetS. The subjects with a smoking habit (heavy and long-time history) had a 1.833-fold higher risk for MetS than their counterparts (none and light smoking), and the subjects that preferred salty taste had a 1.626-fold higher risk for MetS than the comparison cohort. Smoking and alcohol drinking had the highest clustering effect on MetS among the behavior risk factors.

**Conclusions:**

The main finding of this study was that smoking and salty taste preference were the independent determinants of MetS. Smoking and alcohol consumption had the highest clustering effect on southern Chinese MetS.

## 1. Introduction

The metabolic syndrome (MetS) is characterized by a clustering of metabolic disorders, including abdominal obesity, high blood pressure (HBP), high fasting blood glucose, and dyslipidemia (increased triglyceride levels and (or) decreased high-density lipoprotein cholesterol (HDL-C)). It is now becoming a global serious public health challenge that threatened the health care system as it directly contributes to the incidence of type 2 diabetes mellitus (T2DM) and cardiovascular disease (CVD) [1]. The prevalence of MetS has approached 30% in the United States, 21.27% in Asia, 16.04% in Africa, 10.47% in Europe [2], and 24.5% in China [3]. China has undergone a series of remarkable transformations, e.g., rapid economic growth, industrialization, and urbanization, eventually leading to a more severe prevalence of MetS nowadays [4]. The cost of health care and loss of potential economic activity of MetS are in trillions. In consideration of emerging pandemic of MetS, it forces the Chinese government to treat the prevention and treatment of MetS as a crucial task. Given that the present trend is not sustainable, there is an urgent need to identify effective strategies to early intervene and better control MetS. There are certainly some risk factors in the causation of MetS that cannot be changed, but others are capable of being curtailed and corrected. The factual and unmodifiable risks of MetS include gender, age [3], and family history [5]. The acquired and modifiable behavior risk factors of MetS are physical activity (PA), dietary habit, cigarette smoking, alcohol consumption, and sleep duration [6, 7].

PA was showed to play an important role in preventing and mitigating MetS; in some ways, each component of MetS could be favorably intervened by PA. Even though the beneficial effect of PA on MetS was affirmed by numerous studies, PA as a treatment for MetS remained underutilized. The future attention should be paid to PA given the strength of exercise interventions on health outcomes among the MetS individuals [8]. Diet with increased intake of energy-rich foods has long been implicated to be associated with the incidence of MetS. Dietary guidelines for CVD are mainly focused on the fat intake rather than carbohydrate. However, in fact, carbohydrate makes up the large proportion in the Asian diet. Asian food including Chinese food, a typical rice-based diet, in which at least half of the energy is from carbohydrate, consumes low fat when compared with the western food. A growing body of evidence indicated that a high-carbohydrate and low-fat diet might play a significant role in the prevention and management of Asian MetS [9, 10]. In addition, it recently revealed that a higher intake of sodium was positively correlated to MetS components in Chinese [11]. Cigarette smoking is a related risk factor for MetS. Moreover, it was suggested that cigarette smoking could induce the onset of MetS prior to CVD [12, 13]. The current and former smokers presented a greater risk for MetS when compared to nonsmokers [7]. Alcohol consumption was also reported to be positively correlated with MetS; in addition, alcohol consumption less than 40 g/day for men and 20 g/day for women significantly reduced the risk of MetS [14]. Last but not least important, sleep duration has been consistently studied as a risk factor for MetS. There is a U-shaped association between sleep duration and MetS; that is, both too-short and too-long sleep duration are risks of developing MetS [15].

Given the increased prevalence of MetS, guidelines from various professional organizations have called for greater efforts to reduce its behavior risk factors. Clinicians have traditionally evaluated each of the risk factors contributing to MetS on an individual basis. There is evidence, however, that the behavior risk factor influenced each other in a clustered fashion instead of acting independently on the health condition [16, 17]. One behavior risk factor may be involved in the prevalence of another; e.g., alcohol drinking is more likely to partake in cigarette smoking than nondrinking. When two behavior risk factors cluster, the intervention on either one may affect the other one, even though the latter is not directly targeted. Furthermore, the interventions which simultaneously improve the clustered factors are more effective and less costly [18, 19]. The clustering effect of these factors is thus worth treating as a broader entity rather than separately. Incorporating these risk factors as an integral treatment strategy for MetS appears to go a long way toward reducing the adverse health impact of this condition [20].

Previous studies have reported several clustering patterns of behavior risk factors in the general population, such as heavy drinking and smoking, heavy drinking and poor diet, and smoking and physical inactivity which were also clustered [21–23]. However, the clustering effect of behavior risk factors among the southern Chinese MetS populations remains unclear. Thus, the objectives of our study are as follows: (1) to observe the major behavior risk factors of MetS among people with MetS in southern China and (2) to investigate the clustering pattern of these behavior risk factors (PA, dietary habit, cigarette smoking, alcohol consumption, and sleep duration).

## 2. Methods

### 2.1. Design

A cross-sectional study design was applied in this study. The sociodemographic characteristics, family history, and behavior risk factors among southern Chinese people with MetS were first observed. The clustering effect of these behavior risk factors was then explored and analyzed. A STROBE checklist was performed and included as a supplemental file (File S1).

### 2.2. Subjects and Setting

This study used a convenience sampling method to recruit subjects in the Cardiology Clinic, Endocrine Clinic, and Health Management Center of Xiangya Hospital, Central South University, from March to December in 2019. The target population who met the inclusion criteria and signed the informed consent was included in the study. The subjects were aged 18-80 years from all over the country, mostly the southern part of China. The sample size of this study was calculated based on the overall prevalence 24.5% of MetS among the Chinese population [3]. To estimate this order by PASS 15.0 within a 95% confidence limit and a 5% margin of error, a sample size of 285 was required. With a potential attrition rate of 15%, a sample size of 328 was required. We finally collected 594 subjects in the study, in which 62 were not included in the final analysis due to the missing data.

The inclusion criteria were as follows: (a) being aged 18-80 years old; (b) diagnosed as MetS: according to the latest Chinese guideline [24], MetS was determined by the presence of at least 3 of the following parameters: abdominal obesity (WC ≥ 90 cm in men and ≥85 cm in women); fasting blood glucose (FBG) ≥ 6.1 mmol/L, or 2-hour plasma glucose ≥ 7.8 mmol/L after a 75 g oral glucose load, and/or has been diagnosed and treated for diabetes; elevated BP (≥130/85 mmHg and/or has been diagnosed and treated for HBP); fasting blood triglycerides (TG) ≥ 1.7 mmol/L; and fasting blood high-density lipoprotein cholesterol (HDL‐C) < 1.04 mmol/L; (c) being able to read and understand the questionnaire; and (d) providing an informed consent. The exclusion criteria were as follows: (a) being unable to communicate in Mandarin, (b) refusing to participate or not meeting all inclusion criteria, (c) with cardiac function grade III or above, (d) with liver or renal insufficiency, (e) diagnosed with malignant tumors, and (f) severe cardiovascular and cerebrovascular events occurred.

### 2.3. Measures

#### 2.3.1. Demographic and Clinical Characteristics

A demographic sheet was designed to collect the demographic and clinical characteristics of the subjects, including age, gender, level of education, place of residence, and family history of HBP, T2DM, and CVD. Height, weight, and waist circumference (WC) were measured early in the morning after fasting. WC was measured with a tape at the midpoint between the lower margin of the ribs and the iliac crest [25]. Blood pressure (BP) was measured by an Omron electronic sphygmomanometer at a sitting position as the subjects were calmed down at least 5 minute after arriving at the hospital. The value of BP was obtained as the average of two measurements [26]. The information of diagnosis (hypertension and diabetes), medication usage (antihypertensive and antidiabetic pills), and laboratory data (lipid profiles and fasting blood glucose) were all obtained from the electronic medical records and physical examination reports.

#### 2.3.2. Measurement of Behavior Risk Factors

Behavior risk factors (PA, dietary habit, cigarette smoking, alcohol consumption, and sleep duration) were all evaluated based on self-reports from the subjects. International Physical Activity Questionnaire-Short (IPAQ-S) was applied to evaluate the level of PA in this study. The IPAQ-S presents adequate test-retest reliability (Spearman *p* = 0.76) and convergent validity against accelerometer (Spearman *p* = 0.30) and thus is widely used for simple and quick PA assessment in the population surveillance and large-scale studies [27, 28]. The Chinese version of IPAQ-S [29], translated by Macfarlane and colleagues, was adopted in the present study. The IPAQ-S consisted of seven items and provided information on the time spent in the vigorous-intensity activity (aerobic exercise), moderate-intensity activity (leisure cycling), and walking. Subjects were asked to recall the frequency and duration of each activity they performed during the last 7 days. Data from the questionnaire was summed within each item (i.e., vigorous intensity, moderate intensity, and walking) to estimate the total amount of time spent in PA per day. Using the official IPAQ-S scoring protocol, the total PA score was estimated by summing the product of reported time by a PA category and expressed as the total score of MET. The total score of MET was calculated as duration and frequency multiplied by the metabolic equivalent (MET) value. MET values for vigorous-intensity activity, moderate-intensity activity, and walking were 8, 4, and 3.3, respectively (Wolin et al. 2008). The total PA score was divided into three categories: category 1 (low PA level): subjects who did not meet the criteria for category 2 or 3 were put in this category; category 2 (moderate PA level): at least 20 minutes of vigorous-intensity activity per day for 3 or more days per week, or at least 30 minutes of moderate-intensity activity per day for 5 or more days per week, or 5 or more days of any combination of walking, moderate-intensity, or vigorous-intensity activities achieving a minimum total PA of at least 600 MET minutes per week; and category 3 (high PA level): vigorous-intensity activity for at least 3 days achieving a minimum total PA of at least 1500 MET minutes per week or 5 or more days of any combination of walking, moderate-intensity, or vigorous-intensity activities achieving a minimum total PA of at least 3000 MET minutes per week [30].

Other behavior risk factors, i.e., dietary habit, cigarette smoking, alcohol consumption, and sleep duration, were observed by the questionnaire. Dietary habit was evaluated by the intake of vegetables and fruits, soy foods, pickled food, sugary drinks, and taste (salty, moderate, and light) during the last week. Cigarette smoking was classified into 5 categories: none, never smoking; light, <20 cigarettes/day; heavy, ≥20 cigarettes/day and <5 years; long-time history (≥5 years and quit ≤12 months; ≥5 years and quit ≥12 months); and the passive smoking status. Alcohol consumption was divided into 3 categories: none, never drinking; light, <25 g/day in males and <15 g/day in females; and heavy, ≥25 g/day in males and ≥15 g/day in females. Sleep duration was assessed by the average length of sleep during the last week, classified into 3 categories: <6 hours; 6-8 hours; and >8 hours.

### 2.4. Data Analysis

Data analysis was performed using SPSS 25.0 (IBM Corporation Armonk, New York, USA). Descriptive statistics was used to calculate the percentage, mean, and standard deviation. The chi-squared test and *t*-test were performed to compare the sociodemographic characteristics, family history, and behavior risk factors between the subjects with MetS and their counterparts. The stepwise logistic regression analysis was performed to identify the determinants of MetS, including variables for sociodemographic characteristics, family history, and behavior risk factors. The multiple logistic regression analysis was used to analyze the clustering effects of behavior risk factors in MetS. The statistical significance was set at *p* < 0.05 (two-sided tests).

### 2.5. Ethical Consideration

This study design followed the Declaration of Helsinki, which was approved by the ethics committee. The informed written consent forms were obtained from all subjects before participating in the study.

## 3. Results

### 3.1. Sociodemographic Characteristics and Family History

A total of 594 subjects were recruited in the present study, in which 62 sets of data were not eligible. Of the 532 eligible subjects, 43.4% were male and 56.6% were female, and the mean age was 48.4 ± 15.3 years. Approximately 33.3% of the eligible subjects were diagnosed as MetS. It was demonstrated in Table 1 that there was a significant difference in the age, sex, education level, place of residence, and family history of HBP between MetS population and non-MetS population (all *p* < 0.05). The prevalence of MetS increased with age and decreased with education level. The prevalence of MetS in males (46.3%) was significantly higher than that in females (23.3%). Rural people (40.4%) seemed to be more prone to develop MetS than urban people (28.9%). In the MetS group, the subjects with family history of HBP had higher prevalence than those without family history of HBP (41.8% vs. 30.7%). See more details in Table 1.

### 3.2. Behavior Risk Factors of MetS

Table 2 presents the major behavior risk factors of MetS among the subjects in this study. There were significant differences in cigarette smoking (including passive smoking), alcohol consumption, physical activity of self-report, sugar-sweetened drink intake, personal taste, and sleep length (all *p* < 0.05) between MetS and non-MetS. The prevalence of MetS in those who currently smoke heavily (50%) and who used to smoke (71.9% quit <12 months and 63.6% quit ≥12 months, respectively) was higher than that in those who smoke far less (26.6% of nonsmoking and 34.6% of light smoking, respectively). In addition, the passive smokers were more prone to suffer MetS than nonpassive smokers (39.1% vs. 27.2%). It revealed that the prevalence of MetS in heavy drinkers (51.4%) was higher than that in nondrinkers (29.9%) or light drinkers (40.9%). Moreover, the prevalence of MetS in the physical inactivity subjects was higher than that in the physical activity individuals (42.9% vs. 31.7%), but unfortunately, there was no significant difference of MetS prevalence among the diverse exercise intensity and frequency groups. The survey of the diet habit exhibited that the prevalence of MetS in the salty preference group (41.3%) was higher than that in other groups (29.8% of moderate taste and 30.0% of light and others, respectively); however, no sugar-sweetened drinkers seemed to be more prone to MetS in this study (36.9% vs. 21.1%). Last, the survey of sleep duration in the study showed that those who slept 6-8 hours per day (29.3%) had the lowest risk of MetS than other groups (>8 hours 31.7% and <6 hours 44.8%, respectively).

### 3.3. Determinants of MetS

Table 3 mainly reveals that the elderly (≥45 years) were 3.227-3.972 times more likely than younger ones (<45 years) to suffer MetS, and males were 2.093-2.717 times more likely than females to suffer MetS (all *p* < 0.05). In addition, after controlling for risk factors such as gender, age, education, and family history, the subjects with a smoking habit (heavy and long-time history) had a 1.833-fold higher risk for MetS than those nonsmoking and light smoking subjects, and the subjects that preferred salty taste had a 1.626-fold higher risk for MetS than the comparison cohort (all *p* < 0.05).

### 3.4. The Clustering Effects of Behavior Risk Factors in MetS

Table 4 exhibits the clustering effects of behavior risk factors in MetS. After being adjusted for age, gender, education, place of residence, and family history, it implied that smoking and alcohol drinking were highly clustered (OR: 2.452), salty taste and abnormal sleep length were less clustered (OR: 2.342), and physical inactivity and abnormal sleep length were also clustered (OR: 2.104, all *p* < 0.05).

## 4. Discussion

The first finding of this study was that the prevalence of MetS was 33.3%, which was higher than 24.5% that was reported from a meta-analysis of pooled prevalence of MetS among Chinese subjects [3]. The prevalence may vary due to diverse populations of different regions, cultural behaviors, lifestyle habits, and the use of different diagnostic criteria [12, 31, 32]. Under the same criterion of MetS, the prevalence of MetS of the same age group Chinese population [33] was far lower than that of the present study (14.39% vs. 33.3%). One of the primary reasons is that majority of the subjects (73.4%) were from Cardiology Clinic and Endocrine Clinic. Furthermore, dyslipidemia and hypertension accounted for high proportions in the Cardiology Clinic [34], on the other hand, insulin resistance and diabetes occupied a large proportion in the Endocrine Clinic [35].

In addition, the present study further revealed that the prevalence of MetS was associated with age, sex, education level, rural residence and HBP family history. The prevalence of MetS was progressively arising with age growth, larger portion of the male cohort, and the lower level of education, consistent with the findings of previous studies [36, 37]. In particular, a previous study showed that the prevalence of MetS in males was peaking at 40–59 years and then decreasing; however, the prevalence was peaking at the ≥60 years age group in female population [37]. Moreover, it was proved that the prevalence of MetS was negatively correlated with the education background [33, 37]. The prevalence of MetS reported in this study was higher in the rural residents than in the urban residents, which corresponded with the previous survey; this might be due to the different diet habits among the districts [38]. In our study, a family history of HBP was found to be related to MetS. Although the family history of T2DM and CVD in this study was not found to be significantly correlated to the prevalence of MetS, recent studies showed that a positive family history of T2DM [39] as well as CVD [40] was a risk factor for MetS. In the present study, a part of subjects having unknown family history, i.e., 13.2%, 15.4%, and 14.8% of subjects having “unknown” family history of HBP, T2DM, and CVD, respectively, might attribute to the recall bias in the assessment of disease family history. Considering the above factors, the health providers should focus on the middle-age males and postmenopausal females living in the rural place with lower level of education and a family history of HBP, T2DM, and CVD.

After controlling for the factors such as age, gender, education, place of residence, and family history, the main finding of this study was that cigarette smoking and salt taste preference were the independent determinants of MetS. Studies had shown that smoking was an independent risk factor of MetS in Chinese population, especially in males [36, 41]. Similarly, extra salt intake was also considered an independent risk factor of MetS [42]. MetS was believed to be associated with high salt intake through excess reactive oxygen species (ROS) production [43]. Furthermore, studies reported that smoking and a high-salt taste were related to the onset of hypertension among the rural residents compared to the urban residents. In the rural place, the awareness of hypertension prevention-related knowledge is weak, and local people prefer to use the salt method to preserve meat, vegetables, etc., leading to dramatically increased intake of salt and thus making local people more susceptible to hypertension [44, 45]. In the present study, 40.8% of rural subjects were identified as MetS, which was much higher than the prevalence (28.8%) in the urban subjects. A large portion of rural subjects in this study, coupled with their lifestyle habits (smoking and high-salt intake) discussed above, echoes the previous finding—an outbreak of MetS among our subjects in this study.

Furthermore, the clustering effects of the behavior risk factors were analyzed by controlling for age, gender, education, place of residence, and family history. It was established that those with two behavior risk factors had approximately 2.1-2.5 times greater risk of suffering MetS than those with none. Researchers found that lifestyle risk factors were not randomly distributed. However, lifestyle risk factors tend to cluster with other unhealthy behaviors within individuals; in other words, some certain patterns of combined lifestyle risk factors were more common than could be expected based on the prevalence of individual lifestyle risk factor [46]. For instance, smoking and betel quid chewing were discovered to have an interacted effect on increasing risk for MetS, pointing out that incorporating health interventions on these two behaviors into the health program is more cost-effective than the current single-behavior program [36]. In the present study, smoking plus alcohol drinking increased MetS risk 2.5 times more than for those who do not smoke or drink alcohol. In China, the majority of health education programs for MetS emphasize physical activity and healthy diet; however, no specific program was designed for MetS, and no programs focused on both smoking and alcohol consumption. Our study provides suggestion for determining the most effective strategies to reduce MetS risk and a multifaceted approach to promote healthy lifestyle behaviors. Further research is needed on the mechanisms by which multiple behavior risk factors and other related factors cause the occurrence of MetS. Understanding these mechanisms may optimize the health programs in prevention and treatment of MetS.

There are some limitations of this study. First, data were all collected by questionnaire and answered by recall; hence, some may lack objectivity and accuracy, thus threatening the internal validity of the study. Second, when investigating the dietary habits of the subjects, the current study was focused on the taste; however, carbohydrate intake and fat diets were not included, leading to somewhat biased results. Third, the current study evaluated the clustering effects of two behavior risk factors; however, three or more behavior risk factors were not applied in the analysis due to the limited sample. In the future, machine learning analysis will be used to assess the clustering effects of more risk factors and produce more meaningful personal benefits.

## 5. Conclusion

The main strength of this study is that a large-scale regional representative sample was used to investigate the clustering effect of behavior risk factors in southern Chinese MetS after controlling for several potential confounders. Our study displayed high prevalence of MetS in southern China, and the prevalence was associated with age, sex, education level, rural residence, and HBP family history. After adjusting these factors, it was demonstrated that smoking and salty taste preference were the independent determinants of MetS. Furthermore, smoking and alcohol consumption interacted to adversely affect MetS. This study lends support to the emphasis placed on a multiple approach targeting at least two behavior risk factors for the future prevention and intervention programs for MetS in the clinic care and further provides a data evidence to develop efficient prevention and intervention activities for southern Chinese MetS patients.

## Figures and Tables

**Table 1:** Comparison of sociodemographic characteristics and family history between MetS and non-MetS.

		MetS	Non-MetS	*p* value
Variables	*n* = 532	*n* = 177	*n* = 355	
Age, *n* (%)				0.000
18-34 years	126 (23.7)	11 (8.7)	115 (91.3)	
35-44 years	79 (14.8)	19 (24.1)	60 (75.9)	
45-59 years	195 (36.7)	78 (40.0)	117 (60.0)	
≥60 years	132 (24.8)	69 (52.3)	63 (47.7)	
Sex, *n* (%)				0.000
Male	231 (43.4)	107 (46.3)	124 (53.7)	
Female	301 (56.6)	70 (23.3)	231 (76.7)	
Education, *n* (%)				0.002
Primary school	90 (16.9)	38 (42.2)	52 (57.8)	
Junior high school	144 (27.1)	60 (41.7)	84 (58.3)	
Senior high school	71 (13.3)	24 (33.8)	47 (66.2)	
College degree	76 (14.3)	21 (27.6)	55 (72.4)	
Undergraduate and above	151 (28.4)	34 (22.5)	117 (77.5)	
Place of residence, *n* (%)				0.007
Rural	193 (36.3)	78 (40.4)	115 (59.6)	
Urban	339 (63.7)	98 (28.9)	241 (71.1)	
Family history of HBP, *n* (%)				0.014
Yes	196 (36.8)	80 (40.8)	116 (59.2)	
Unknown	70 (13.2)	23 (32.9)	47 (67.1)	
No	266 (50.0)	74 (27.8)	192 (72.2)	
Family history of T2DM, *n* (%)				0.087
Yes	45 (8.5)	21 (46.7)	24 (53.3)	
Unknown	82 (15.4)	30 (36.6)	52 (63.4)	
No	405 (76.1)	126 (31.1)	279 (68.9)	
Family history of CVD, *n* (%)				0.121
Yes	91 (17.1)	38 (41.8)	53 (58.2)	
Unknown	79 (14.8)	28 (35.4)	51 (64.6)	
No	362 (68.1)	111 (30.7)	251 (69.3)	

MetS: metabolic syndrome; HBP: high blood pressure; T2DM: type 2 diabetes mellitus; CVD: cardiovascular disease.

**Table 2:** Comparison of behavior risk factors between MetS and non-MetS.

		MetS	Non-MetS	*p* value
Variables	*n* = 532	*n* = 177	*n* = 355	
Smoking, *n* (%)				0.000
None	399 (75.0)	106 (26.6)	293 (73.4)	
Light	26 (4.9)	9 (34.6)	17 (65.4)	
Heavy	64 (12.0)	32 (50.0)	32 (50.0)	
Long-time history (quit ≤12 months)	32 (6.0)	23 (71.9)	9 (28.1)	
Long-time history (quit >12 months)	11 (2.1)	7 (63.6)	4 (36.4)	
Passive smoking, *n* (%)				0.004
Yes	289 (54.3)	113 (39.1)	176 (60.9)	
No	243 (45.7)	66 (27.2)	177 (72.8)	
Alcohol, *n* (%)				0.007
None	402 (75.6)	120 (29.9)	282 (70.1)	
Light	93 (17.4)	38 (40.9)	55 (59.1)	
Heavy	37 (7.0)	19 (51.4)	18 (48.6)	
PA (self-report), *n* (%)				0.045
No	84 (15.8)	36 (42.9)	48 (57.1)	
Yes	448 (84.2)	142 (31.7)	306 (68.3)	
PA intensity, *n* (%)				0.236
Low	185 (34.8)	63 (34.1)	122 (65.9)	
Moderate	305 (57.3)	105 (34.4)	200 (65.6)	
High	42 (7.9)	9 (21.4)	33 (78.6)	
PA (MET)	1116.9 ± 1069.9	1009.2 ± 935.4	1136.2 ± 1070.3	0.180
Fruit-vegetable intake, *n* (%)				0.059
<5 days/week	99 (18.6)	25 (25.3)	74 (74.7)	
≥5 days/week	433 (81.4)	153 (35.3)	280 (64.7)	
Soy food intake, *n* (%)				0.207
No	112 (21.1)	43 (38.4)	69 (61.6)	
Yes	420 (78.9)	135 (32.1)	285 (67.9)	
Pickled food intake, *n* (%)				0.709
<3 days/week	400 (75.2)	135 (33.8)	264 (66.2)	
≥3 days/week	132 (24.8)	42 (32.1)	89 (67.9)	
Sugar-sweetened drink intake, *n* (%)				0.001
Yes	123 (23.1)	26 (21.1)	97 (78.9)	
No	409 (76.9)	151 (36.9)	258 (63.1)	
Taste preferences, *n* (%)				0.017
Salty	137 (25.8)	59 (43.1)	78 (56.9)	
Moderation	188 (35.3)	56 (29.8)	132 (70.2)	
Light and others	207 (38.9)	62 (30.0)	145 (70.0)	
Sleep length/day, *n* (%)				0.008
<6 hours	125 (23.5)	56 (44.8)	69 (55.2)	
6-8hours	328 (61.7)	96 (29.3)	232 (70.7)	
>8 hours	79 (14.7)	25 (31.7)	54 (68.4)	

MetS: metabolic syndrome; PA: physical activity; MET: metabolic equivalent of task. Cigarette smoking was classified into 5 categories: none, never smoking; light, <20 cigarettes/day; heavy, ≥20 cigarettes/day and <5 years; long-time history (≥5 years and quit ≤12 months; ≥5 years and quit ≥12 months); and the passive smoking status. Alcohol consumption was divided into 3 categories: none, never drinking; light, <25 g/day in males and <15 g/day in females; and heavy, ≥25 g/day in males and ≥15 g/day in females.

**Table 3:** Logistic regression analysis of determinants of MetS by a stepwise method.

Variables	Model 1 (sociodemographic characteristics)				Model 2 (sociodemographic characteristics+family history)				Model 3 (sociodemographic characteristics+family history+behavior risk factors)			
	*B*	OR	95% CI	*pv*alue	*B*	OR	95% CI	*p* value	*B*	OR	95% CI	*p* value
Age (0, <45 years; 1, ≥45 years)	1.379	3.972	2.406-6.558	0.000	1.370	3.937	2.372-6.536	0.000	1.171	3.227	1.874-5.555	0.000
Gender (0, female; 1, male)	1.000	2.717	1.826-4.044	0.000	0.995	2.705	1.814-4.035	0.000	0.738	2.093	1.274-3.439	0.004
Education (0, college degree and above;1, senior high school and below)	0.240	1.271	0.780-2.070	0.336	0.267	1.306	0.800-2.133	0.285	0.159	1.172	0.706-1.946	0.539
Place of residence (0, urban; 1, rural)	0.005	1.005	0.635-1.589	0.984	0.032	1.032	0.650-1.640	0.893	0.048	1.049	0.649-1.695	0.846
Family history of T2DM (0, no; 1, yes+not sure)					0.144	1.155	0.666-2.002	0.608	0.165	1.179	0.672-2.068	0.566
Family history of CVD (0, no; 1, yes+not sure)					-0.229	0.796	0.470-1.347	0.395	-0.239	0.787	0.455-1.361	0.392
Family history of HBP (0, no; 1, yes+not sure)					0.442	1.555	0.997-2.477	0.063	0.384	1.469	0.910-2.372	0.116
Smoking (0, none+light; 1, heavy+long-time history)									0.606	1.833	1.027-3.271	0.040
Alcohol (0, none+light; 1, heavy)									0.137	1.147	0.523-2.517	0.732
PA of intensity (0, moderate+high; 1, low)									0.395	1.485	0.638-3.456	0.359
Intake of vegetable and fruit (0, ≥5 days/week; 1, <5 days/week)									-0.296	0.744	0.425-1.301	0.300
Intake of pickled food (0, <3days/week; 1, ≥3days/week)									-0.080	0.923	0.573-1.489	0.743
Intake of sugar-sweetened drink (0, no; 1, yes)									-0.247	0.781	0.443-1.378	0.394
Soy foods (0, yes; 1, no)									0.118	1.126	0.693-1.829	0.632
Taste preference (0, others; 1, salty)									0.486	1.626	1.030-2.568	0.037
Sleep length (0, 6-8 h; 1, <6 h or >8 h)									0.437	1.548	0.972-2.465	0.066

MetS: metabolic syndrome; PA: physical activity; OR: odd ratios; CI: confidence intervals; T2DM: type 2 diabetes mellitus; CVD: cardiovascular disease; HBP: high blood pressure.

**Table 4:** The clustering effects of behavior risk factors in MetS assessed by multiple logistic regression analysis^a^.

Variables					*B*	OR	95% CI	*p* value
Smoking (0, never+light; 1, heavy+long-time history)	Alcohol (0, never+light; 1, heavy)	PA (0, ≥ moderate intensity; 1, low intensity)	Taste (0, other tastes; 1, salty taste)	Sleep (0, ≥6 h; 1, <6 h or >8 h)				
1	1	0	0	0	0.897	2.452	1.016-5.917	0.046
1	0	1	0	0	-0.146	0.864	0.420-1.777	0.691
1	0	0	1	0	0.040	1.041	0.485-2.236	0.917
1	0	0	0	1	-0.094	0.910	0.382-2.167	0.831
0	1	1	0	0	-0.382	0.682	0.161-2.896	0.604
0	1	0	1	0	0.477	1.612	0.576-4.512	0.366
0	1	0	0	1	-0.551	0.577	0.137-2.396	0.449
0	0	1	1	0	0.533	1.704	0.905-3.209	0.099
0	0	1	0	1	0.744	2.104	1.096-4.041	0.025
0	0	0	1	1	0.851	2.342	1.150-4.768	0.019

PA: physical activity; MetS: metabolic syndrome; BRF: behavior risk factors; OR: odd ratios; CI: confidence intervals. Unhealthy behaviors include 5 items of smoking, alcohol abuse, lack PA, like salty taste, and abnormal sleep duration. There were 3 missing data. ^a^Adjusted for age, gender, education, place of residence, and family history. ^∗^Used multiple regression model.

## Data Availability

The data used to support the findings of this study are included within the article.

## Supplementary Materials

Supplementary Materials STROBE Statement—Checklist of items that should be included in reports of cross-sectional studies.
